# Thermal and Acidic Treatments of Gluten Epitopes Affect Their Recognition by HLA-DQ2 *in silico*

**DOI:** 10.3389/fnut.2021.647750

**Published:** 2021-05-19

**Authors:** Jihui Gao, Haolan Du, Zekun Zhou, Zhongxin Liang, Hongrui Liang, PeiAo Zhang, Xue Wei, Shujun Liu, Linglin Fu, Yanbo Wang, Huilian Che, Wentong Xue, Fengjiao Xin, Dong Yang

**Affiliations:** ^1^Beijing Key Laboratory of Functional Food From Plant Resources, College of Food Science and Nutritional Engineering, China Agricultural University, Beijing, China; ^2^Institute of Food Science and Technology, Chinese Academy of Agricultural Sciences, Beijing, China; ^3^Food Safety Key Laboratory of Zhejiang Province, School of Food Science and Biotechnology, Zhejiang Gongshang University, Hangzhou, China

**Keywords:** gluten, epitope, immunogenecity, peptide, HLA-DQ2

## Abstract

Celiac disease (CD) is a prevalent disorder with autoimmune features. Dietary exposure of wheat gluten (including gliadins and glutenins) to the small intestine activates the gluten-reactive CD4^+^ T cells and controls the disease development. While the human leukocyte antigen (HLA) is the single most important genetic factor of this polygenic disorder, HLA-DQ2 recognition of gluten is the major biological step among patients with CD. Gluten epitopes are often rich in Pro and share similar primary sequences. Here, we simulated the solution structures changes of a variety of gluten epitopes under different pH and temperatures, to mimic the fermentation and baking/cooking processes. Based on the crystal structure of HLA-DQ2, binding of differently processed gluten epitopes to DQ2 was studied *in silico*. This study revealed that heating and pH change during the fermentation process impact the solution structure of gluten epitope. However, binding of differently treated gluten epitope peptide (GEP) to HLA-DQ2 mainly depended on its primary amino acid sequence, especially acidic amino acid residues that play a pivotal role in their recognition by HLA-DQ2.

## Introduction

Wheat is one of the most widely grown cereal crop, and bread made with wheat flour is one of the oldest staple food since the Neolithic era of human history (1, 2). Flour-based staple food supplies not only carbohydrates but also proteins, which takes 8–15% of the wheat kernel weight, to human diet (3). Among wheat proteins, 85–90% is gluten, and intolerance to it leads to an autoimmune disease called celiac sprue (4). Gluten includes glutenins and gliadins, and glutenins are catalyzed by related enzymes and/or oxidants to form a cohesive network as the structural basis of dough (5). Besides gluten from wheat, similar prolamin proteins such as hordeins from rye and secalins from barley could also trigger this inflammation (6). Celiac disease (CD) affects ~1% of the world's population, and it has been suggested that the increasing prevalence of CD over the past decades is partially due to the inadvertently changed immunoreactivity of wheat during breeding (7–10). Undiagnosed disease and poor diet compliance often lead to increased morbidity and mortality (11, 12). However, studies of wheat cultivars over 120 years found no trends in relative or absolute CD-active peptides, indicating a relatively evolutional stability of the CD epitopes (13).

As suggested by its name, prolamin proteins are rich in the amino acid residues proline and glutamine. For example, glutenins often contain repetitive sequences in its central domain, such as the GQQ repeat, the PGQGQQ repeat, and the GYYPTSL(P)QQ repeat (14). The ineffective cleavage of gastrointestinal enzymes to prolamin proteins at sites before and after proline or glutamine often ends up with large CD active peptides, which were absorbed into the lamina propria (15). And they are often resistant to processing by the luminal and brush-border enzymes. As a result, they are transported to the mucosal epithelium in the form of polypeptides, deamidated by tissue transglutaminase (TG2), and recognized predominately by the CD4^+^ T-cells. T-cell recognition is relying mostly by the HLA-DQ2 molecule, preferentially of negatively charged residues at the anchor positions of P4, P6, and P7 and controls the CD development (16–18).

Different processing changes the epitope conformation, which in turn may alter their recognition by HLA-DQ2 (19). To review the post-harvest journey of prolamin proteins, they go through different treatments with various combinations of pH and temperatures depending on the food to make. During the sourdough fermentation process in leavened dough preparation, microbial acidification could bring the pH down to ~3.0–4.5 (20). After being endowed with a certain shape, the dough is then usually steamed/baked at temperatures of 100°C or higher (5). In some cases, the dough is boiled (at 100°C) without fermentation during the cooking of dumplings, spaghettis, and noodles. Eventually, all these deli would be consumed and delivered to the fasted stomach, where the pH is ~2 (21). Considering that the prolamins deep in the deli could enter the gut without sufficient digestion in the stomach, it is possible that the conformation of an epitope at pH 3.0–4.5 is maintained even after passing through the stomach (22). Thus, there are typically two thermal conditions and three pH conditions among different treatment scenarios that a gluten epitope could possibly undergo. It would be enlightening to determine which factor, or combination of them, impact the epitope conformation and subsequent allergenicity more profoundly with a possible molecular mechanism provided.

## Materials and Methods

### Homology Comparison and Structure Generation

The six gluten epitope homologous comparison was conducted with the Multiple Sequence Alignment tool-Clustalw Omega in the European Bioinformatics Institute of the European Molecular Biology Laboratory (EMBL-EBI) (23). And the three-dimensional structures of these peptides were generated with PyMol (24).

### Different Thermal and pH Treatments of the Gluten Epitope Peptides

Different pH and acidic treatments of each of the above gluten epitope peptides (GEPs) were performed with the BIOVIA Discovery Studio software V16.1.0. Based on the CHARMm36 molecular mechanics and molecular dynamics (MD) force field engine, MD was performed in the DS standard dynamics cascade. All systems were solvated in an ~33 × 28 × 59 Å^3^ orthorhombic box with a minimum clearance of 7 Å using the explicit periodic boundary water model and neutralized by the addition of sodium cation and chloride anion to an ionic concentration of 0.3326 (unpublished data). For the acidic treatment, the pH was set at 2.0 and 4.5, respectively. Initially, the system underwent two energy minimization steps: 1,000 steps of steepest descent minimization and 2,000 steps of conjugate gradient minimization with the adopted Newton–Raphson algorithm (25). The following three steps of heating, equilibration, and production were performed afterwards. The whole system was heated from 50 K to target temperature in 4 ps without constraints, and then the equilibrium step was run at target temperature for 20 ps without constraints. The following production step was run at different target temperature and pressure of 1.0 for 200 ps with typed NPT and no constraints. As for the native state, acidic treatment simulations, the target temperature was set at 298.15 K, and the pH was set at 7.5. As for the thermal treatment, and thermal treatment simultaneously combined with acidic treatment, the target temperature was set at 373.15 K. The electrostatic parameter was set to automatic, which recognizes the periodic environment and used the particle mesh Ewald (PME) electrostatic calculation (26). Among the 100 conformations generated, the one in the solvation boundary with the lowest total energy was selected for the subsequent study.

### Immunogenicity Assessment as Recognition by HLA-DQ2

The selected conformation of each GEP after different treatments was applied to molecular docking to the epitope presenting groove of the HLA-DQ2 X-ray crystal structure (PDB ID: 1S9V) where the original GEP1 in the published structure was removed (27). The HLA-DQ2 receptor was further prepared in an ~72 × 60 × 97 Å^3^ orthorhombic box with a minimum clearance of 7 Å *via* the aforementioned MD simulation protocols, except that the pH for protonation was set at pH 7.5, the ionic strength was set at 0.2, and the target temperature for MD production step was set at 310.15 K (28, 29). To evaluate the HLA-DQ2 recognition of differently treated GEPs, molecular docking was performed with the DS CDOCKER module between the native, differently treated GEPs and HLA-DQ2. Both the GEP and DQ2 were prepared at pH 7.5 and ionic strength of 0.2, and the epitope presenting groove was selected as the binding site with the radius set at 22.6 Å. CDOCKER is a grid-based docking mechanism operated by the CDOCKER algorithm (30). Of over 1,000 dynamic steps, 10 random conformations were generated for the initial ligand conformation at a temperature of 1,000 K accompanied with 10 orientations. The following simulated annealing was performed with 2,000 steps heating to a target temperature of 700 K and 5,000 steps cooling to a target temperature of 300 K. Among the 10 GEP-DQ2 binding conformations generated, the complex with the lowest CDOCKER energy was subjected to the Calculate Binding Energies module equipped in the DS, and subsequent association constant calculation was performed. For the GEP-DQ2 interaction analysis, LigPlot was used to analyze the hydrophobic interactions, and PyMol was used to analyze the hydrogen bonding (31).

## Results

### Gluten Epitope Diversity and Rationale in This Study

**Figure 1:**
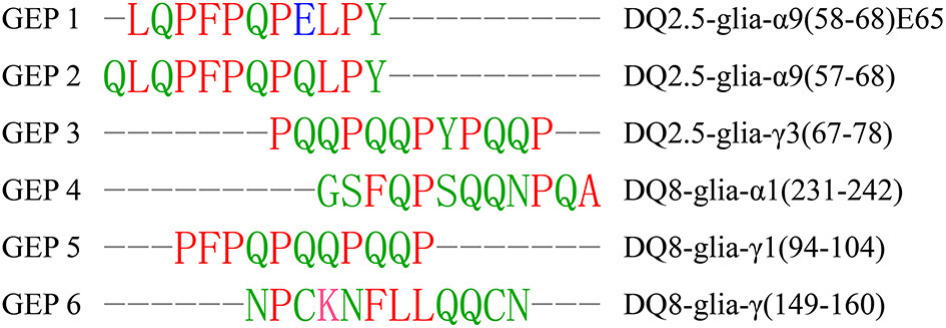
Sequence alignment of the selected gluten epitope peptides (GEPs). The six selected GEPs were chosen among the reported gluten epitopes with maximum primary sequence diversity, and aligned with the European Bioinformatics Institute of the European Molecular Biology Laboratory (EMBL-EBI) multiple sequence alignment tool for sequence comparison. The leftmost lane is the number of the GEP, the middle is its sequence, and the rightmost lane is the number of the amino acid residues in each corresponding GEP. The rightmost lane is the epitope with the most similar amino acid sequences. Red indicates amino acid residues L, P, F, and A; green indicates amino acid residues Q, Y, G, S, N, and C; blue indicates amino acid residue E; magenta indicates amino acid residue K.

Most of the gluten epitope identified so far are sequences in the gliadins, and among the 61 identified gluten epitopes summarized in this study, 42 of them are recognized by the HLA-DQ2 molecule (Supplementary Table 1). The minimal length required for T-cell recognition is nine amino acids (4). Thus, we selected five peptides (GEP2–6) ranging from 11 to 12 amino acids long, which maximally represent the sequence diversity among all the gluten epitopes summarized (Figure 1). Most of these peptides are the common fragments of gluten epitopes identified previously, and epitopes with the most similar amino acid sequences are listed (32). Additionally, another peptide (LQPFPQPELPY, GEP1), which has been reported to bind to DQ2, was studied in parallel as an indicator of the computational accuracy (27). GEP2–6 are very diversified in their primary sequences except GEP1. Among these six GEPs, GEP1, GEP2, GEP3, and GEP5 have been identified as recognized by DQ2 (33). GEP4, GEP5, and GEP6 are identified as recognized by DQ8 (34, 35). So far, GEP4 and GEP6 are identified as solely recognized by HLA-DQ8. Studying their *in silico* recognition by DQ2 may reveal the molecular basis of recognition specificity. There is an acidic glutamic acid (E) in GEP1 and a basic lysine (K) in GEP6, while the rest of the amino acid residues are either aromatic or aliphatic. Specifically, glutamine (Q) and proline (P) are always present in these epitopes, while glutamine takes up 16.7–50% and proline takes up 8.3–45.5% of the total amino acid residues.

In this study, we simulated the conformation of each above GEP under different pH and temperature treatments to mimic the fermentation, cooking, and digestion processes in reality in a simplified module. GEP1 has been co-crystallized with HLA-DQ2 at a pH of buffer mixture (2 μl of 25 mM Tris–HCl pH 8.0 with 2 μl of 50 mM sodium acetate, pH 3.5) and room temperature in previous studies (27). The same GEP1–DQ2 complex was simulated at pH 7.5 and 25°C. Results show that the DQ2 conformations between the one obtained from X-ray diffraction and *in silico* simulation were nearly the same with a root mean square deviation (RMSD) of 1.292 Å. This suggested a relatively robust computational result that sufficiently reflected the experimental one. The RMSD between the GEP1 in the crystal structure and the simulated structure is 2.315 Å; this slight difference in structure is probably due to the change in pH during crystallization (8.0 vs. 7.5) and the difference of “room temperature” and 25°C.

**Figure 2:**
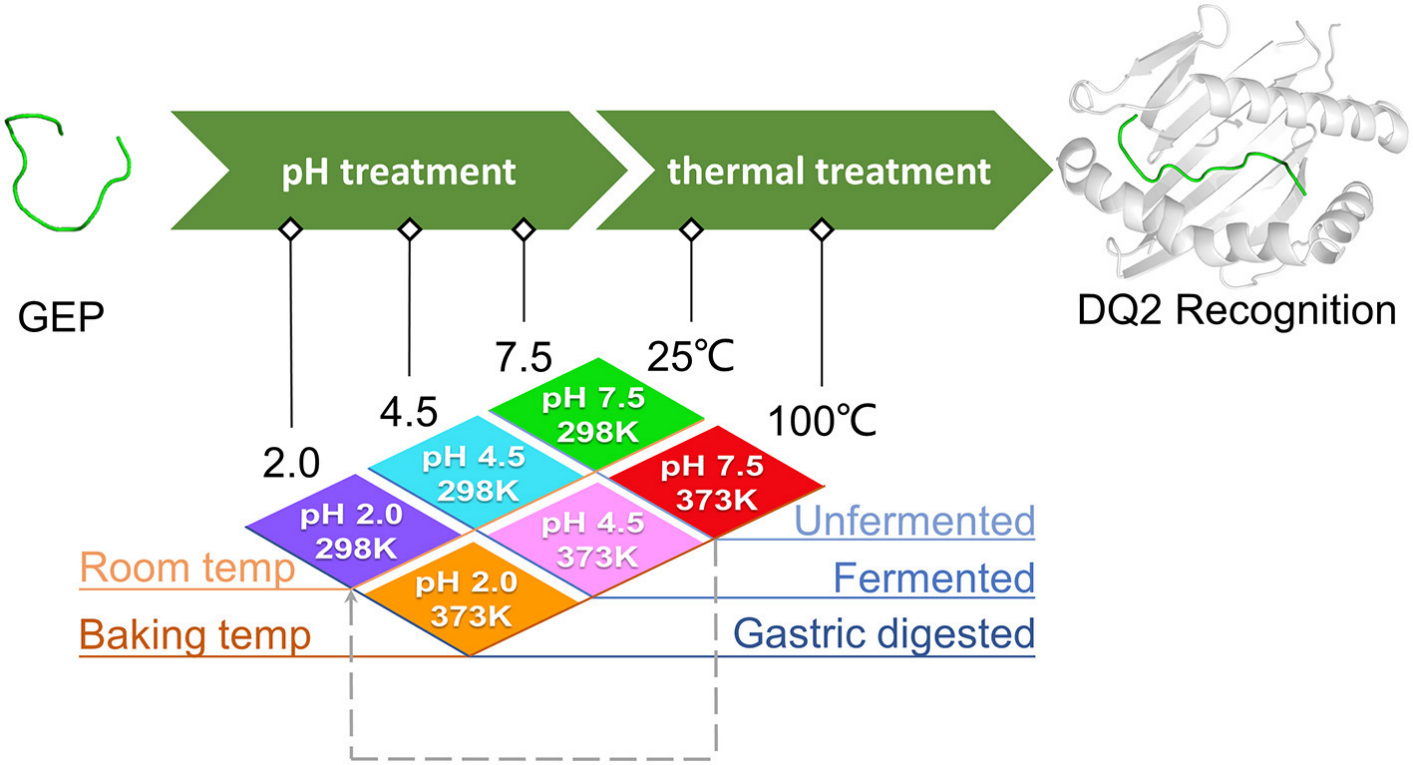
Study rationale and scheme of different treatments on the gluten epitope peptides (GEPs). The native gluten protein (pH 7.5 at 25°C), with GEP sequences on it, went through low pH treatment (pH 4.5 during bacterial fermentation and pH 2.0 during gastric digestion in the fasted stomach) and thermal treatment (100°C during boiling/steaming) under different circumstances. The pH and temperature conditions in all scenarios were simplified into a combination of temperatures (298.15 and 373.15 K) and pH (2.0, 4.5, and 7.5). Each GEP went through a treatment of one of the above combinations. The dashed arrow represents the case where the dough was treated at pH 7.5 and 100°C before taken in the diet (pH 2.0 and 25°C). The conformation of GEPs after the above seven treatments was applied for recognition by HLA-DQ2, and the molecular docking parameters generated served as indicators for their immunogenicity.

As mentioned above, for different pH conditions, these situations could be (1) the untreated/unleavened dough at physiological pH 7.5; (2) the microbial leavened dough at pH 4.5; and (3) prolamins in the stomach treated at pH 2.0. For different temperature conditions, the scenarios are simplified into two cases: (1) the uncooked dough at room temperature of 25°C and (2) the cooked dough at 100°C (baking temperatures above 100°C are simplified to this temperature). In this theoretical study, the pH and temperature conditions were simplified and systemized. Three different pH values were applied in this study: pH 7.5 represents the native structure of untreated GEPs, pH 4.5 represents mild acidic treatment where the sourdough fermentation potentially affects the recognition of GEPs, and pH 2.0 represents intensified acid treatment where the gluten was digested in a fasted stomach before entering the small intestine. Two temperatures were applied in this study: 25°C represents the GEPs at their native temperature, and 100°C represents the heat treatment. Different combinations of these pH and temperatures represent scenarios of eating different food (wheat flour based). For example, treatment at pH 4.5 and 100°C simplifies the situation where the steamed bread (fermented) was consumed, while treatment at pH 7.5 and 100°C simplifies the situation where an unleavened bread was cooked and consumed (Figure 2). Additionally, the treatment of pH 7.5 and 100°C, followed by treatment of pH 2.0 and 25°C, was studied to investigate the gastric digestion (pH 2.0) of a cooked dough with GEP in it (Figure 2, dashed line).

Following different combinations of treatment, each GEP was applied to the recognition by HLA-DQ2 *in silico*. Their recognition, specifically at the peptide-presenting groove of the DQ2 heterodimer interface as previously reported, triggers the presentation of these GEPs to T-cells in the small intestine (4). Thus, it is an indicator to evaluate the immunogenicity of a GEP conformation by measuring its recognition by HLA-DQ2.

### Heat Treatment to the Conformation of Gluten Epitopes and Their Recognition by HLA-DQ2

**Figure 3:**
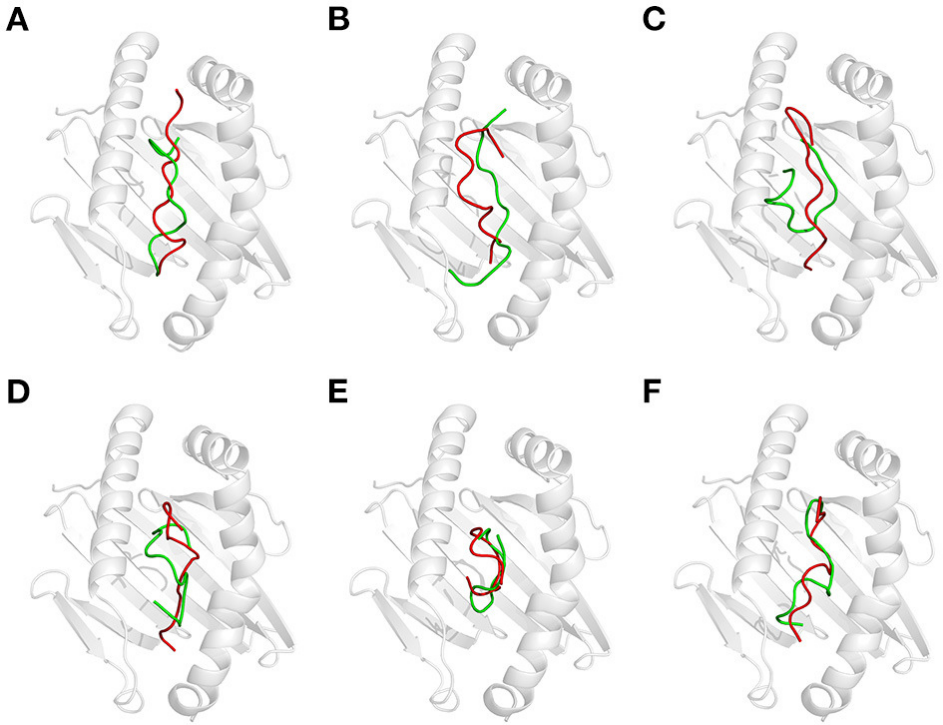
Effect of thermal treatment on the recognition of gluten epitope peptides (GEPs). In each panel, the white cartoon representation presents the HLA-DQ2 with the epitope presenting groove facing outwards. The ribbon diagram represents each GEP. The green ones represent native GEPs at pH 7.5 and 25°C, and the red ones represent the thermal treated one at pH 7.5 and 100°C. **(A–F)** GEP1–GEP6, respectively.

Generally, heat treatment at 100°C substantially influenced the conformation of each GEP (Figure 3). For GEP1, recognition by DQ2 rendered a conformational change of this peptide by an RMSD of 2.833 Å (Figure 3A). The binding energy calculated from the CDOCK pose with the best score was −18.06 kJ/mol. Heat treatment deviated this structure from its native conformation by 3.519 Å, and binding to DQ2 further deviated its conformation from the heat-treated one for 2.918 Å (Table 1). Compared with the native DQ2, the number of hydrogen bonds that formed between GEP1 and DQ2 did not change, while the number of amino acid residues involved in their hydrophobic interactions increased from eight to nine in GEP1 and from 16 to 20 in DQ2 (Table 1). Binding energy of the heat-treated GEP1–DQ2 complex was calculated as 25.70 kJ/mol that leads to an ~10^−8^-fold decrease in the binding affinity.

**Table 1:** Binding parameters of differently treated GEP to HLA-DQ2.

**GEP**	**Treatment^a^**	**RMSD^b^ (Å)**		**H-bond^c^**	**Hydrophobic interactions^d^**		**-CDOCKER_Energy (kJ/mol)**	**Binding energy (kJ/mol)**	**Fold change^e^**
		**Apo-**	**Complex**		**GEP**	**DQ2**			
GEP1	Native	-	2.833	6	8	16	91.417	−18.06	1
	Thermal	3.519	2.918	6	9	20	91.991	25.704	−−−
	Acidic	3.412	5.013	5	8	15	91.523	−32.917	+
	Thermal→acidic	3.036	2.187	7	9	15	77.441	−71.362	+++
	Thermal + acidic	4.948	6.751	8	7	12	100.05	−110.197	+++++
GEP2	Native	-	2.49	8	7	12	97.661	−35.801	1
	Thermal	4.473	3.865	7	8	16	103.333	−37.52	+
	Acidic	2.922	4.599	8	8	13	93.062	−146.546	++++++
	Thermal→acidic	3.666	5.046	12	5	13	97.45	−114.467	+++++
	Thermal + acidic	4.717	2.439	5	11	11	90.677	−36.869	+
GEP3	Native	-	7.464	4	8	9	105.68	−107.904	1
	Thermal	3.317	2.632	9	9	13	105.278	−83.393	−−
	Acidic	2.49	4.413	12	8	16	89.354	−100.185	−
	Thermal→acidic	4.118	5.554	9	8	12	90.234	−146.7	++
	Thermal + acidic	3.221	3.889	10	11	16	95.405	−110.683	+
GEP4	Native	-	6.021	6	5	7	130.191	−15.707	1
	Thermal	1.31	5.877	13	8	11	136.997	−109.147	+++++
	Acidic	3.41	3.543	4	9	19	124.675	−18.95	+
	Thermal→acidic	1.499	4.285	8	9	13	119.475	−77.955	++++
	Thermal + acidic	3.534	5.23	6	9	13	126.646	−51.78	++
GEP5	Native	-	6.981	5	8	11	89.723	−168.754	1
	Thermal	4.268	5.808	7	10	11	88.878	−87.384	−−−−−
	Acidic	3.185	2.751	5	10	14	76.625	−2.767	−−−−−−−−−−
	Thermal→acidic	3.286	5.543	5	8	12	77.887	−122.648	−−−
	Thermal + acidic	2.049	6.827	6	9	9	79.793	−105.359	−−−−
GEP6	Native	-	6.593	5	10	17	154.2	56.631	1
	Thermal	4.301	5.436	6	8	13	148.007	−77.677	++++++++
	Acidic	3.853	4.018	4	8	14	144.691	46.745	+
	Thermal→acidic	5.459	4.883	10	9	11	144.543	−25.624	+++++
	Thermal + acidic	4.011	5.576	7	7	13	143.858	−26.142	+++++

a *Treatment conditions, native stands for the gluten epitope peptide (GEP) conformation at pH 7.5 and 25°C; thermal treatment stands for the GEP conformation at pH 7.5 and 100°C; acidic treatment stands for the GEP conformation at pH 2 and 25°C; thermal→acidic treatment stands for the GEP conformation at pH 7.5 and 100°C and then simulated at pH 2.0 and 25°C; thermal + acidic treatment stands for the GEP conformation at pH 2.0 and 100°C*.

b *RMSD is the root mean square deviation; apo- is the RMSD between conformations of differently treated GEPs and the corresponding native GEP; DQ2 complex is the RMSD between the conformations of differently treated GEPs in the GEP-DQ2 complex and the corresponding native apo-GEPs*.

c *Number of hydrogen bonds is the number of hydrogen bonds that formed between native and differently treated GEPs and the DQ2*.

d *Number of residues involved in hydrophobic interactions represents the numbers of amino acid residues in GEP/DQ2 involved in their interactions after different treatments*.

e *Fold change is the change of association constant of one particular treated GEP binding to DQ2 compared with that of the corresponding native GEP binding to DQ2. + indicates a fold increase <10^3^; ++ indicates a fold increase between 10^3^ and 10^7^; +++ indicates a fold increase between 10^7^ and 10^10^; ++++ indicates a fold increase between 10^10^ and 10^13^, and so on. – indicates a fold decrease with the same magnitude as the + sign*.

For GEP2, its recognition by DQ2 rendered a structural change of 2.49 Å. Heat treatment deviated its conformation from the native one for 4.473 Å, and the DQ2 bound conformation was greatly compressed compared with that of the native one with an RMSD of 3.865 Å (Figure 3B). The number of hydrogen bonds that formed between GEP2 and DQ2 decreased from eight to seven, while there are more (eight compared with seven) amino acid residues in GEP2 and more (16 compared with 12) amino acid residues in DQ2 involved in their hydrophobic interactions (Table 1). The binding energy calculated from the best CDOCKER pose was −35.801 kJ/mol for native GEP2 binding to DQ2 and −37.520 kJ/mol for the thermal denatured, and this subsequently led to an increase of binding affinity of ~2-fold.

For GEP3, binding to DQ2 dramatically altered the conformation of this peptide with an RMSD of 7.464 Å (Figure 3C), and heat treatment changed its conformation with an RMSD of 3.317 Å (Table 1). The heat-treated GEP3 was recognized by DQ2 with structural adjustment of 2.632 Å. The GEP3–DQ2 interaction was enhanced as the hydrogen bonds increased from four to nine between them, and one more amino acid residue in GEP3 and four more amino acid residues in DQ2 are involved in the hydrophobic interactions between them. The binding energy calculated was −107.90 kJ/mol for the native GEP3 binding to DQ2 and −83.39 kJ/mol for the heat-treated one, and this caused an ~10^−5^-fold decrease in binding affinity.

For GEP5, DQ2 recognition leads to an intensive structural change with an RMSD of 6.981 Å (Figure 3E). Heat treatment changed the unbound GEP5 structure with an RMSD of 4.268 Å, and binding of the heat-treated GEP5 bound to DQ2 deviated from its unbound conformation with an RMSD of 5.808 Å. The number of hydrogen bonds that formed in the GEP5–DQ2 complex increased from five to seven, and the number of amino acid residues in GEP5 involved in hydrophobic interactions increased from 8 to 10, while the number of amino acid residues in DQ2 involve in their hydrophobic interactions did not change (Table 1). The binding energy was calculated as −168.75 kJ/mol and decreased to −87.38 kJ/mol as the GEP5 was thermal treated. This led to a very significant decrease in their binding affinity.

Although right now GEP4 and GEP6 are only reported to bind to HLA-DQ8, they are still recognized by HLA-DQ2 here *in silico* with structural adjustment (RMSD of 6.021 and 6.593 Å, respectively) to fit the peptide-presenting groove (Figures 3D,F). Heat treatment changed the conformation of GEP6 more significantly (RMSD of 4.301 Å) than GEP4 (RMSD of 1.310 Å), and subsequent binding to DQ2 leads to conformational rearrangement of 5.877 and 5.436 Å, respectively. After heat treatment, GEP4 increased its interaction with DQ2, as seven more hydrogen bonds were formed and seven more amino acid residues were involved in their hydrophobic interactions. On the other hand, GEP6 formed one more hydrogen bond with DQ2, but six less amino acid residues were involved in their hydrophobic interactions (Table 1). For both GEP4 and GEP6, thermal treatment caused a significant decrease in their binding energy and consequently a large increase in their binding affinity to DQ2.

### Acidic Treatment to the Conformation of Gluten Epitopes and Their Recognition by HLA-DQ2

**Figure 4:**
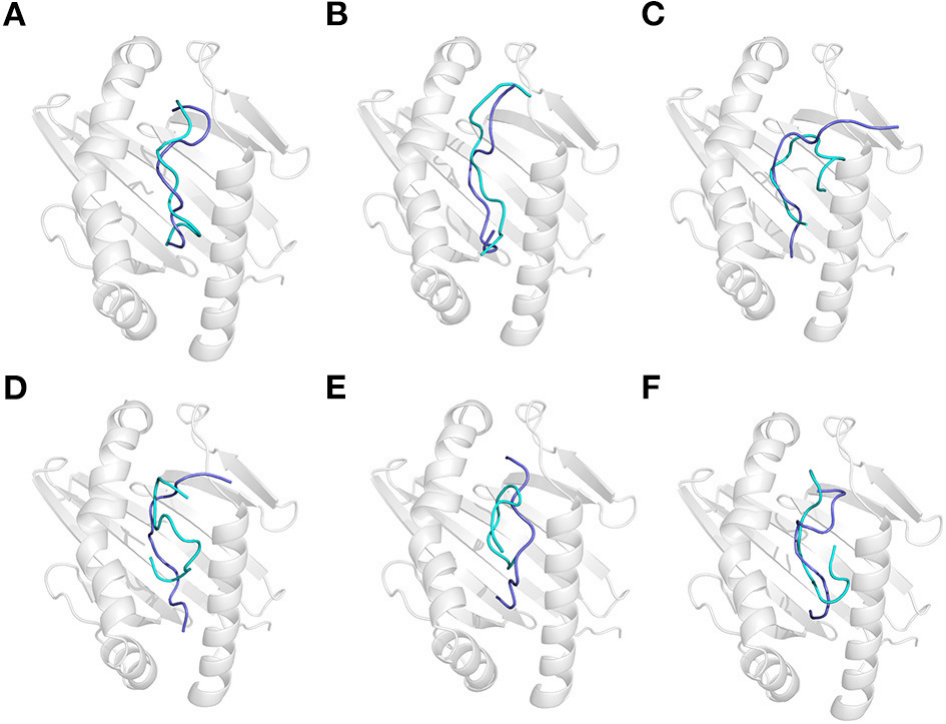
Effect of acidic treatment on the recognition of gluten epitope peptides (GEPs). In each panel, the white cartoon representation presents the HLA-DQ2 with the epitope presenting groove facing outwards. The ribbon diagram represents each GEP. The cyan ones represent native GEPs at pH 4.5 and 25°C, and the slate ones represent the thermal treated one at pH 2.0 and 25°C. **(A–F)** GEP1–GEP6, respectively.

Acidic treatment mimics the bacterial fermentation in the dough at pH 4.5 and gastric digestion in a fasted stomach at pH 2.0. It is found that the conformation of all GEPs at pH 4.5 is identical with that at pH 7.5 (Supplementary Table 2). Each GEP was compared with its folding at pH 4.5 and pH 2.0, while the former was noted as the native and the latter does impact the structure of all GEPs (Figure 4). For GEP1, acidic treatment rendered a structural change with an RMSD of 3.412 Å, and recognition by HLA-DQ2 deviated its structure with an RMSD of 5.013 Å (Figure 4A). The number of both the hydrogen bonds and the amino acid residues involved in their hydrophobic interactions between GEP1 and DQ2 decreased slightly (Table 1). However, the binding energy after acidic treatment decreased to −32.92 kJ/mol, indicating an ~10^2^-fold increased binding affinity.

For GEP2, acidic treatment deviated its structure with an RMSD of 2.922 Å. When this acid-treated peptide bound to DQ2, its conformation was changed with an RMSD of 4.599 Å (Figure 4B). Compared with that of the native one, the interaction between the acid-treated GEP2 and DQ2 was merely more intensive by only one more amino acid residue, in both GEP2 and DQ2, involved in their hydrophobic interaction (Table 1). The binding energy dramatically decreased to −146.55 kJ/mol, suggesting an astounding increase of ~10^18^-fold in binding affinity.

For GEP3, acidic treatment changed its conformation by an RMSD of 2.490 Å, and further binding to HLA-DQ2 changed its structure by another RMSD of 4.413 Å (Figure 4C). Acid-treated GEP3 binds with DQ2 with eight more hydrogen bonds, and seven more amino acid residues in DQ2 are involved in their hydrophobic interactions (Table 1). The binding energy of acid-treated GEP3 to DQ2 slightly increased to −100.19 kJ/mol, which leads to a slight decrease of binding affinity.

For GEP5, acidic treatment changed the peptide conformation by an RMSD of 3.185 Å (Figure 4E), and binding to DQ2 leads to a structural adjustment with an RMSD of 2.751 Å (Table 1). Compared with the native structure, acidic treatment strengthened the interaction between GEP5 and HLA-DQ2 by increasing their hydrophobic interactions where two more amino acid residues in GEP5 and three more amino acid residues in DQ2 are involved in their recognition. The binding energy of acid-treated GEP5 to DQ2 dramatically increased to −2.77 kJ/mol, which led to a significant decrease of ~10^−28^-fold.

For GEP4 and GEP6, acidic treatment deviated their structures from each corresponding one with RMSD of 3.410 and 3.853 Å, while DQ2 binding changed their structures from the apo-one for 3.543 and 4.018 Å, respectively (Figures 4D,F). For GEP4, less hydrogen bonds were formed while significantly more hydrophobic interactions were involved after acidic treatment. For GEP6, hydrogen bonding and hydrophobic interactions were both weakened in acid-treated GEP6–DQ2 complex (Table 1). For both GEP4 and GEP6, there is a slight decrease in their binding energies to DQ2, which leads to an increase in their binding affinities.

### Combination of Thermal and Acidic Treatment to the Conformation of Gluten Epitopes and Their Recognition by HLA-DQ2

**Figure 5:**
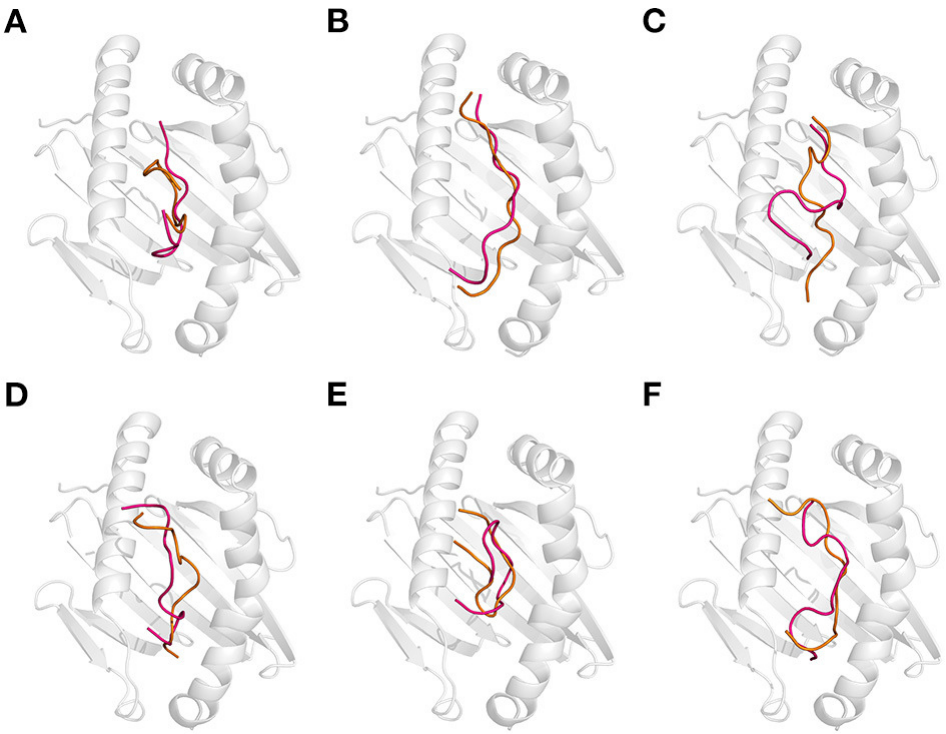
Effect of the combination of thermal and acidic treatments on the recognition of gluten epitope peptides (GEPs). In each panel, the white cartoon representation presents the HLA-DQ2 with the epitope presenting groove facing outwards. The ribbon diagram represents each GEP. The hot pink ones represent the thermal treated one at pH 7.5 and 100°C followed by acidic treatment at pH 2.0 and 25°C. The orange ones represent the simultaneous thermal and acid-treated ones at pH 2.0 and 100°C. **(A–F)** GEP1–GEP6, respectively.

To mimic the combination of thermal and acidic treatment, the thermal treatment at 100°C (cooking) was followed by acidic treatment at pH 2.0 (gastric digestion). As a theoretical study, the situation where the GEPs were treated at pH 2.0 and 100°C was also investigated. That is, the thermal and acidic treatments were undertaken simultaneously. For GEP1, thermal treatment followed by acidic treatment leads to a structural change from the native one with an RMSD of 3.036 Å, and recognition by DQ2 leads to a change with an RMSD of 2.187 Å (Figure 5A). Thermal treatment followed by acidic treatment altered the GEP1–DQ2 interaction by increasing one hydrogen bond. One more amino acid residue in GEP1 and one less amino acid residue in DQ2 were involved in their hydrophobic interactions. The binding energy between GEP1 and DQ2 after such treatment decreased to −71.36 kJ/mol, which subsequently leads to a large increase of their binding affinity of ~10^8^-fold. Once the thermal and acidic treatments were applied simultaneously, the apo-GEP1 structure deviated more severely with an RMSD of 4.948 Å, and docking to the DQ2 peptide-presenting groove resulted in a severe compressed structural change of 6.751 Å. There are two more hydrogen bonds that formed, while one less amino acid residue in GEP1 and four less amino acid residue in DQ2 were involved in their hydrophobic interactions (Table 1). And the corresponding binding energy decreased to −110.19 kJ/mol, which led to a very large increase of their binding affinity of ~10^15^-fold.

For GEP2, thermal treatment followed by acidic treatment changed its structure with an RMSD of 3.666 Å, and binding to DQ2 leads to a structural change of 5.046 Å (Figure 5B). There were four more hydrogen bonds that formed, and two less amino acid residues in GEP2 and one more amino acid residue in DQ2 were involved in their hydrophobic interactions. This led to a large decrease of their binding energy to −114.47 kJ/mol, which corresponds to an ~10^13^-fold increase of their binding affinity. When the thermal and acidic treatments were applied simultaneously, there is a structural change with an RMSD of 4.717 Å, and recognition by DQ2 changed its conformation by 2.439 Å. There are three less hydrogen bonds that formed, while four more amino acid residues in GEP2 and one less amino acid residue in DQ2 were involved in their hydrophobic interactions (Table 1). The corresponding binding energy only slightly decreased to −36.87 kJ/mol, and the binding affinity almost did not change.

For GEP3, thermal treatment followed by acidic treatment deviated the peptide structure from its native form with an RMSD of 4.118 Å, and binding to DQ2 leads to a structural change with an RMSD of 5.554 Å (Figure 5C). The GEP3–DQ2 interaction was intensified by forming five more hydrogen bonds, and three more amino acid residues in DQ2 were involved in their hydrophobic interactions. This treatment leads to a decrease of their binding energy to −146.70 kJ/mol that caused an ~10^6^-fold increase of their binding affinity. Simultaneous thermal and acidic treatments deviated the peptide from its native structure with an RMSD of 3.221 Å, and subsequent binding to DQ2 leads to a conformational change with an RMSD of 3.889 Å. The GEP3–DQ2 interaction was strengthened by six more hydrogen bonds, while three more amino acid residues in GEP3 and seven more amino acid residues in DQ2 were involved in their hydrophobic interactions. This treatment also leads to a decrease of binding energy to −110.68 kJ/mol that translated to a slight increase of their binding affinity.

For GEP5, acidic treatment after thermal treatment of this peptide changed it structure with an RMSD of 3.286 Å, and subsequent binding to DQ2 leads to a structural change of 5.543 Å (Figure 5E). There is little enhancement of the GEP5–DQ2 interactions by one more amino acid residue in DQ2 involved in their interactions after such treatment. This treatment led to an increase to their binding energy to −122.65 kJ/mol that translated to an ~10^−8^-fold decrease of binding affinity. Once the thermal and acidic treatments were applied at the same time, the peptide structure was changed with an RMSD of 2.049 Å, and subsequent binding to DQ2 leads to a structural change of 6.827 Å. The number of hydrogen bonds that formed in between them increased by one. One more amino acid residue in GEP5 and two less amino acid residues in DQ2 were involved in their interactions. This treatment caused an increase in their binding energy to −105.36 kJ/mol, which corresponds to a 10^−11^-fold decrease in their binding affinity.

For GEP4 and GEP6, acid treatment following thermal treatment deviated these epitopes from their native structures with RMSD of 1.499 and 5.459 Å, respectively. Subsequent binding to DQ2 adjusted their conformations with RMSD of 4.285 and 4.883 Å, respectively (Figures 5D,F). There is a significant enhancement of GEP4–DQ2 interaction with the hydrogen bonds increased by two, and the number of amino acid residues increased by 10 in the GEP4–DQ2 complex. And the binding energy increased to −77.96 kJ/mol, which corresponded to an ~10^10^-fold increase in their binding affinity. There are five more hydrogen bonds that formed in the GEP6–DQ2 complex, while one less amino acid residue in GEP6 and six less amino acid residues in DQ2 were involved in their hydrophobic interactions. The binding energy decreased to −25.62 kJ/mol, which corresponded to an ~10^13^-fold increase in their binding affinity.

When the thermal and acidic treatments were applied simultaneously, the conformations of these peptides were deviated from their native forms with RMSD of 3.534 and 4.011 Å, and recognition of these treated peptides by DQ2 changed their structures with RMSD of 5.230 and 5.576 Å (Figures 5D,F). For GEP4, the hydrogen bonding remained the same, while the hydrophobic interactions were enhanced by four more amino acid residues in GEP4 and six more amino acid residues in DQ2. This treatment leads to a decrease of the binding energy to −51.78 kJ/mol that corresponded to an ~10^6^-fold increase in their binding affinity. For GEP6, two more hydrogen bonds formed, while a total of seven less amino acid residues were involved in the GEP6–DQ2 hydrophobic interactions. And this led to a decrease of binding energy to −26.14 kJ/mol, which corresponded to an ~10^13^-fold increase in their binding affinity.

## Discussion

Heat treatment, which almost certainly undermines the native structure of a protein, does not necessarily decrease the allergenicity of a protein allergen (36). For example, roasting decreased the allergenicity of hazelnut while increasing the IgE binding of two major allergens, Ara h 1 and Ara h 2, in peanuts (37, 38). Even in the same food, heat treatment exerts different allergenicity effects depending on the protein component. For example, boiling treatment lowered the IgE binding to shrimp extracts but enhanced the IgE binding to the purified TM from boiled shrimps (39). It is consistent in this study that different GEPs responded differently to heat treatment in exhibiting their binding to HLA-DQ2.

For GEP1 and GEP2, there is one extra Q in the N-terminus of GEP2, and the E8 is changed into Q in GEP2. Thus, these two epitopes exhibited a similar binding behavior to HLA-DQ2 after acidic treatment: thermal treatment followed by acidic treatment and simultaneously thermal and acidic treatments. The difference is that they corresponded differently to the thermal treatment at physiological pH. For both GEP1 and GEP2, one more amino acid residue in the peptide and four more amino acid residues in the DQ2 are involved in the hydrophobic interaction after thermal treatment, indicating an exposure of hydrophobic residues after thermal treatment of these epitopes. However, the conformation of exposed GEP1 and GEP3 is obviously different. The thermal treated and DQ2 bound GEP1 peptide is compressed compared with its native one, while that of the GEP2 is more severely compressed. Consistent with previous studies, this result indicated that the pivotal role of one acidic amino acid residue in the gluten epitope could profoundly impact its immunogenicity during food processing (17, 18).

For these six GEPs studied here, GEP1, GEP2, GEP3, and GEP5 are known to be recognized by DQ2. Their responses to different treatments on exhibiting DQ2-mediated immunogenicity are vastly different. For GEP1 and GEP3, some treatments decrease their binding to HLA-DQ2, while other treatments increase their binding. For GEP2 and GEP4, all treatments increased their binding. On the other hand, all treatments decreased the binding of GEP5. Considering the sequence similarity between GEP2 and GEP5, a little variance in primary sequence does lead to dramatically different DQ2 recognition.

GEP4 and GEP6 are reported to be only recognized by DQ8. It is not clear that the uncertainty of recognition of these two epitopes by HLA-DQ2 is due to the lack of evidence or not. However, *in silico* study here not only revealed that they are recognized by HLA-DQ2 but also indicated that thermal and acidic treatments both sensitized their recognition. Interestingly, thermal treatment alone intensified the recognition of these two GEPs most, while any form of acidic treatment, following the thermal treatment or in a simultaneous combination with thermal treatment, weakened this effect. It is noticed the FxxxQxN sequence is in both GEP4 and GEP6 that formed an intra-chain kink structure. Neither thermal treatment nor acidic treatment disrupted this structure. For GEP4 and GEP6, all treatments increased their binding, and this suggested a possible role of kink structures in the HLA-DQ2-induced immunogenicity.

Of course, this study was performed by placing all the GEPs in an infinitely diluted solution. In reality, the GEPs are in the cellular conditions and the molecular crowding effect, chaperons, and pH buffering that survive the GEPs from the thermal and acidic treatments (40–42). What could happen *in silico* does not necessarily mean it could happen in reality. However, a theoretical study like this offers a structural and mechanistic insight into what could happen as the molecular basis of different food processing methods to decrease the allergenicity. This is potentially helpful in the *in vitro* mutagenesis and breeding strategies to obtain wheat species with reduced amount of gluten epitopes for the prevention of CD. For example, breeding between the diploid *Triticum monococcum* and hexaploid wheat could potentially generate a cultivar with reduced immune response due to the higher digestibility of gluten proteins in the former (43).

## Conclusions

In this study, computational simulation of thermal, acidic, and thermal treatment followed by further acidic treatment, and simultaneous thermal and acidic treatment of GEPs with each subsequent binding to HLA-DQ2 were performed. Results show that the amino acid sequences of each GEP profoundly impact their recognition by HLA-DQ2, especially the acidic amino acid residues dramatically change the binding characteristics of a GEP. The peptide conformation, such as a kink, is stable and conserved during different treatments and binding to DQ2. Our results indicated GEP4 and GEP6 still bind to HLA-DQ2; however, this kink structure might be structurally characteristic of HLA-DQ8 recognized gluten epitopes.

## Data Availability Statement

The raw data supporting the conclusions of this article will be made available by the authors, without undue reservation.

## Author Contributions

DY and FX designed the study. JG, HD, ZZ, ZL, HL, and PZ performed the experiment. HD, JG, XW, SL, LF, YW, HC, WX, FX, and DY analyzed the data. DY wrote the paper. ZZ, HD, and JG contributed equally to this work. All authors read and approved the final manuscript.

## Conflict of Interest

The authors declare that the research was conducted in the absence of any commercial or financial relationships that could be construed as a potential conflict of interest.
