# Supplementary material for: Thermal and Acidic Treatments of Gluten Epitopes Affect Their Recognition by HLA-DQ2 in silico
Source: Front Nutr. 2021 May 19;8:647750. doi: 10.3389/fnut.2021.647750 (PMC8169964; doi:10.3389/fnut.2021.647750)
Supplement: Supplementary file 1 [file Table_1.docx]

Supplementary Material

for

Thermal and Acidic treatments of gluten epitopes affect their recognition by HLA-DQ2 *in* *silico*

Jihui Gao^1†^, Haolan Du^2†^, Zekun Zhou^1†^, Zhongxin Liang^1^, Hongrui Liang^1^, PeiAo Zhang^1^, Wei Xue^2^, Shujun Liu^2^, Linglin Fu^3^, Yanbo Wang^3^, Huilian Che^1^, Wentong Xue^1^, Fengjiao Xin^2*^ and Dong Yang^1*^

^1^ Beijing Key Laboratory of Functional Food from Plant Resources, College of Food Science & Nutritional Engineering, China Agricultural University, Beijing, China

^2^ Institute of Food Science and Technology, Chinese Academy of Agricultural Sciences, Beijing, China

^3^ Food Safety Key Laboratory of Zhejiang Province, School of Food Science and Biotechnology, Zhejiang Gongshang University, Hangzhou, China

^†^**Equal contributions**

*** Correspondence:**Dong Yang or Fengjiao Xin
dyang@cau.edu.cn or 2002hongzhi30@163.com

Supplementary Table1 List of Gluten Epitopes

| Number | Sequences | Origin/Location |  | Antigen/Name | References |
| --- | --- | --- | --- | --- | --- |
| 1 | LQLQPFPQPQLPYPQPQLPYPQPQLPYPQPQPF | α-2 gliadin/56-88 |  | - | (1) |
| 2 | FLQPQQPFPQQPQQPYPQQPQQPFPQ | g-5 gliadin/26-51 |  | - | (1) |
| 3 | QQPQQQYPSGQGSFQPSQQNPQAQG | α-1 gliadin/198-222 |  | DQ8 | (2) |
| 4 | QQXSQPQXPQQQQXPQQPQQF | 5 gluten/unknown |  | - | (3) |
| 5 | QPQPFPQQSEQSQQPFQPQPF | 21 gluten/unknown |  | - | (3) |
| 6 | HQPQQTFPQPQQTYPHQPQQ | M36999 γ-gliadin/41-60 |  | DQ2/DQ8 | (4) |
| 7 | IHSVAHSIIMQQEQQQGVPI | M36999 γ-gliadin/201-220 |  | DQ2 | (4) |
| 8 | LGIIQPQQPAQLEGIRSLVL | M36999 γ-gliadin/231-250 |  | DQ2 | (4) |
| 9 | LQPQQPFPQQPQQPYPQQPQ | γ-5 gliadin/60-79 |  | DQ2-γ-Ⅴ | (5) |
| 10 | PFPQPQQTFPQQPQLPFPQQ | M36999 γ-gliadin/71-90 |  | DQ2/DQ8 | (4) |
| 11 | PQQPFPQPQQPQQPFPQSQQ | M36999 γ-gliadin/91-110 |  | DQ2/DQ8 | (4) |
| 12 | PQQPFPQPQQQFPQPQQPQQ | M36999 γ-gliadin/111-130 |  | DQ2 | (4) |
| 13 | PQQPFPQSQQPQQPFPQPQQ | M36999 γ-gliadin/101-120 |  | DQ2/DQ8 | (4) |
| 14 | QFPQPQQPQQSFPQQQQPAI | M36999 γ-gliadin/121-140 |  | DQ2/DQ8 | (4) |
| 15 | QFPQTQQPQQPFPQPQQTFP | M36999 γ-gliadin/61-80 |  | DQ2/DQ8 | (4) |
| 16 | QQPFCQQPQRTIPQPHQTFH | M36999 γ-gliadin/21-40 |  | DQ2 | (4) |
| 17 | QQPQLPFPQQPQQPFPQPQQ | M36999 γ-gliadin/81-100 |  | DQ2/DQ8 | (4) |
| 18 | QQQQPPFSQQQQSPFSQQQQ | 156 glutenin/40-59 |  | - | (3) |
| 19 | QQTYPHQPQQQFPQTQQPQQ | M36999 γ-gliadin/51-70 |  | DQ2 | (4) |
| 20 | QSFLQQQMNPCKNFLLQQCN | M36999 γ-gliadin/141-160 |  | DQ8 | (4) |
| 21 | SFPQQQQPAIQSFLQQQMNP | M36999 γ-gliadin/131-150 |  | DQ8 | (4) |
| 22 | TIPQPHQTFHHQPQQTFPQP | M36999 γ-gliadin/31-50 |  | DQ2 | (4) |
| 23 | WPQQQPFPQPQQPFCQQPQR | M36999 γ-gliadin/11-30 |  | DQ2/DQ8 | (4) |
| 24 | LGQQQPFPPQQPYPQPQPF | α-gliadin/31-49 |  | - | (1) |
| 25 | QLQPFPQPQLPYPQPQS | α-gliadin/57-73 |  | - | (1) |
| 26 | QPQQPQQSFPQQQRPF | γ-30 gliadin/138-153 |  | DQ2 | (6) |
| 27 | GSFQPSQQNPQAQGS | AJ133612 α-gliadin/231-245 |  | DQ8 | (4) |
| 28 | PFPQPQLPYPQPQLP | AJ133612 α-gliadin/61-75 |  | DQ2 | (4) |
| 29 | PQQPQQSFPQQQRPF | γ-1 gliadin/123-137 |  | DQ2 | (7) |
| 30 | QAQGSVQPQQLPQFE | AJ133612 α-gliadin/241-255 |  | DQ2/DQ8 | (4) |
| 31 | QLPYPQPQLPYPQPQ | AJ133612 α-gliadin/66-80 |  | DQ2 | (4) |
| 32 | QPQLPYPQPQLPYPQ | AJ133612 α-gliadin/71-85 |  | DQ2 | (4) |
| 33 | QQPPFSQQQQQPLPQ | 17 glutenin/46-60 |  | - | (3) |
| 34 | VQGQGIIQPQQPAQL | γ-30 gliadin/222-236 |  | DQ2 | (3, 8) |
| 35 | YLQLQPFPQPQLPYP | AJ133612 α-gliadin/56-70 |  | DQ2 | (4) |
| 36 | YPQPQLPYPQPQPFR | AJ133612 α-gliadin/76-90 |  | DQ2 | (4) |
| 37 | YPSGQGSFQPSQQNP | AJ133612 α-gliadin/226-240 |  | DQ8 | (4) |
| 38 | PQPQLPYPQPQLPY | α-2 gliadin/62-75 |  | DQ2 | (1, 9) |
| 39 | QQPFPQQPQQPFPQ | γ-2 gliadin/56-69 |  | DQ2 | (7) |
| 40 | FPQQPQQPYPQQP | γ-5 gliadin/66-78 |  | DQ2-γ-Ⅲ | (5) |
| 41 | GQQGYYPTSPQQS | unknown |  | DQ8–glut-1 | (10) |
| 42 | LGQQQPFPPQQPY | α-gliadin/31-43 |  | - | (1) |
| 43 | QQPQQSFPEQERP | unknown |  | DQ2–glia-γ1 | (10) |
| 44 | SGEGSFQPSQENP | unknown |  | DQ8–glia-α1 | (10) |
| 45 | FSQPQQQFPQPQ | γ-5 gliadin/102-113 |  | DQ2-γ-Ⅳ | (5) |
| 46 | QGYYPTSPQQS | glt04 724–734 |  | DQ8 | (11) |
| 47 | QPQQSFPEQER | unknown |  | DQ2–glia-γ1 | (12) |
| 48 | PQPELPYPQP | unknown |  | DQ2–glia-αⅠⅠ | (12) |
| 49 | QPFPQPELPY | unknown |  | DQ2–glia-αⅠ | (12) |
| 50 | IIQPQQPAQ | γ-5 gliadin/228-236 |  | DQ2-γ-Ⅱ | (3) |
| 51 | PFPQPELPY | unknown |  | DQ2.5-glia-α-1a | (13) |
| 52 | PFPQPQLPY | α-9 gliadin/60-68 |  | DQ2-α-Ⅰ | (9) |
| 53 | PFPQPQLPY | α-2 gliadin/61-69 |  | DQ2-α-Ⅰ | (14) |
| 54 | PFSEQEQPV | unknown |  | DQ2.2-glut-L1 | (13) |
| 55 | PQPQLPYPQ | α-2 gliadin/62-70 |  | DQ2-α-Ⅱ | (9) |
| 56 | PQPQLPYPQ | α-2 gliadin/70-78 |  | DQ2-α-Ⅱ | (14) |
| 57 | PQQSFPQQQ | γ-5 gliadin/115-123 |  | DQ2-γ-I | (6) |
| 58 | PYPEQEEPF | avenin/33-41 |  | - | (14) |
| 59 | PYPQPQLPY | α-2 gliadin/67-75 |  | DQ2-α-Ⅲ | (5) |
| 60 | PYPQPQLPY | α-2 gliadin/75-83 |  | DQ2-α-Ⅲ | (14) |
| 61 | QXPQQPQQF | 5 gluten/unknown |  | - | (8) |

Supplementary Table 2 Binding parameters of native and mild acidic treated GEP to HLA-DQ2

| GEP | ^a^Treatment | ^b^RMSD (Å) | | ^c^H-Bond | ^d^Hydrophobic interactions | | -CDOCKER | Binding | ^e^Fold Change |
| --- | --- | --- | --- | --- | --- | --- | --- | --- | --- |
|  |  |  |  |  |  |  | Energy | Energy |  |
|  |  | Apo- | Complex |  | GEP | DQ2 | (kJ/mol) | (kJ/mol) |  |
| GEP1 | pH4.5/25℃ | 0 | 2.833 | 6 | 8 | 16 | 91.417 | -18.060 | 1 |
|  | pH7.5/25℃ | 0 | 2.833 | 6 | 8 | 16 | 91.417 | -18.060 | 1 |
|  | pH4.5/100℃ | 3.519 | 2.918 | 6 | 9 | 20 | 91.991 | 25.704 | --- |
|  | pH7.5/100℃ | 3.519 | 2.918 | 6 | 9 | 20 | 91.991 | 25.704 | --- |
| GEP2 | pH4.5/25℃ | 0 | 2.490 | 8 | 7 | 12 | 97.661 | -35.801 | 1 |
|  | pH7.5/25℃ | 0 | 2.490 | 8 | 7 | 12 | 97.661 | -35.801 | 1 |
|  | pH4.5/100℃ | 4.473 | 3.865 | 7 | 8 | 16 | 103.333 | -37.520 | + |
|  | pH7.5/100℃ | 4.473 | 3.865 | 7 | 8 | 16 | 103.333 | -37.520 | + |
| GEP3 | pH4.5/25℃ | 0 | 7.464 | 4 | 8 | 9 | 105.680 | -107.904 | 1 |
|  | pH7.5/25℃ | 0 | 7.464 | 4 | 8 | 9 | 105.680 | -107.904 | 1 |
|  | pH4.5/100℃ | 3.317 | 2.632 | 9 | 9 | 13 | 105.278 | -83.393 | -- |
|  | pH7.5/100℃ | 3.317 | 2.632 | 9 | 9 | 13 | 105.278 | -83.393 | -- |
| GEP4 | pH4.5/25℃ | 0 | 6.021 | 6 | 5 | 7 | 130.191 | -15.707 | 1 |
|  | pH7.5/25℃ | 0 | 6.021 | 6 | 5 | 7 | 130.191 | -15.707 | 1 |
|  | pH4.5/100℃ | 1.310 | 5.877 | 13 | 8 | 11 | 136.997 | -109.147 | +++++ |
|  | pH7.5/100℃ | 1.310 | 5.877 | 13 | 8 | 11 | 136.997 | -109.147 | +++++ |
| GEP5 | pH4.5/25℃ | 0 | 6.981 | 5 | 8 | 11 | 89.723 | -168.754 | 1 |
|  | pH7.5/25℃ | 0 | 6.981 | 5 | 8 | 11 | 89.723 | -168.754 | 1 |
|  | pH4.5/100℃ | 4.268 | 5.808 | 7 | 10 | 11 | 88.878 | -87.384 | ----- |
|  | pH7.5/100℃ | 4.268 | 5.808 | 7 | 10 | 11 | 88.878 | -87.384 | ----- |
| GEP6 | pH4.5/25℃ | 0 | 6.593 | 5 | 10 | 17 | 154.200 | 56.631 | 1 |
|  | pH7.5/25℃ | 0 | 6.593 | 5 | 10 | 17 | 154.200 | 56.631 | 1 |
|  | pH4.5/100℃ | 4.301 | 5.436 | 6 | 8 | 13 | 148.007 | -77.677 | ++++++++ |
|  | pH7.5/100℃ | 4.301 | 5.436 | 6 | 8 | 13 | 148.007 | -77.677 | ++++++++ |

a Treatment conditions, native stands for the GEP conformation at pH 7.5, 25℃; thermal treatment stands for the GEP conformation at pH 7.5, 100℃; acidic treatment stands for the GEP conformation at pH2, 25℃; thermal→acidic treatment stands for the GEP conformation at pH 7.5, 100℃, then simulated at pH 2.0, 25℃; thermal+acidic treatment stands for the GEP conformation at pH 2.0, 100℃.

b RMSD is the root mean square deviation; apo- is the RMSD between conformations of differently treated GEPs and the corresponding native GEP; DQ2 complex is the RMSD between the conformations of differently treated GEPs in the GEP-DQ2 complex and the corresponding native apo-GEPs.

c Number of hydrogen bonds is the number of hydrogen bonds formed between native and differently treated GEPs and the DQ2.

d Number of residues involved in hydrophobic interactions represents the numbers of amino acid residues in GEP/DQ2 involved in their interactions after different treatment.

e Fold change is the change of association constant of one particular treated GEP binding to DQ2 compare to that of the corresponding native GEP binding to DQ2. + indicates a fold increase<10^3^, ++ indicates a fold increase between 10^3^ and 10^7^; +++ indicates a fold increase between 10^7^ and 10^10^; ++++ indicates a fold increase between 10^10^ and 10^13^, and so on. – indicates a fold decrease with the same magnitude as the + sign.

References

1. M. G. Gänzle, J. Loponen and M. Gobbetti: Proteolysis in sourdough fermentations: mechanisms and potential for improved bread quality. *Trends in Food Science and Technology*, 19(10), 513-521 (2008)

2. Y. Van De Wal, Y. M. Kooy, P. A. Van Veelen, S. A. Peña, L. M. Mearin, Ø. Molberg, K. E. Lundin, L. M. Sollid, T. Mutis and W. E. Benckhuijsen: Small intestinal T cells of celiac disease patients recognize a natural pepsin fragment of gliadin. *Proceedings of the National Academy of Sciences*, 95(17), 10050-10054 (1998)

3. W. Vader, Y. Kooy, P. van Veelen, A. de Ru, D. Harris, W. Benckhuijsen, S. Peña, L. Mearin, J. W. Drijfhout and F. Koning: The gluten response in children with celiac disease is directed toward multiple gliadin and glutenin peptides. *Gastroenterology*, 122(7), 1729-1737 (2002)

4. S. Tollefsen, H. Arentz-Hansen, B. Fleckenstein, Ø. Molberg, M. Ráki, W. W. Kwok, G. Jung, K. E. Lundin and L. M. Sollid: HLA-DQ2 and-DQ8 signatures of gluten T cell epitopes in celiac disease. *The Journal of Clinical Investigation*, 116(8), 2226-2236 (2006)

5. H. Arentz–Hansen, S. N. Mcadam, Ø. Molberg, B. Fleckenstein, K. E. Lundin, T. J. Jørgensen, G. Jung, P. Roepstorff and L. M. Sollid: Celiac lesion T cells recognize epitopes that cluster in regions of gliadins rich in proline residues. *Gastroenterology*, 123(3), 803-809 (2002)

6. H. Sjöström, K. Lundin, O. Molberg, R. Körner, S. McAdam, D. Anthonsen, H. Quarsten, O. Norén, P. Roepstorff and E. Thorsby: Identification of a gliadin T-cell epitope in coeliac disease: general importance of gliadin deamidation for intestinal T-cell recognition. *Scandinavian journal of immunology*, 48, 111-115 (1998)

7. L. W. Vader, D. T. Stepniak, E. M. Bunnik, Y. M. Kooy, W. De Haan, J. W. Drijfhout, P. A. Van Veelen and F. Koning: Characterization of cereal toxicity for celiac disease patients based on protein homology in grains. *Gastroenterology*, 125(4), 1105-1113 (2003)

8. D. Stepniak, L. W. Vader, Y. Kooy, P. A. van Veelen, A. Moustakas, N. A. Papandreou, E. Eliopoulos, J. W. Drijfhout, G. K. Papadopoulos and F. Koning: T-cell recognition of HLA-DQ2-bound gluten peptides can be influenced by an N-terminal proline at p-1. *Immunogenetics*, 57(1-2), 8-15 (2005)

9. H. Arentz-Hansen, R. Körner, Ø. Molberg, H. Quarsten, W. Vader, Y. M. Kooy, K. E. Lundin, F. Koning, P. Roepstorff and L. M. Sollid: The intestinal T cell response to α-gliadin in adult celiac disease is focused on a single deamidated glutamine targeted by tissue transglutaminase. *The Journal of Experimental Medicine*, 191(4), 603-612 (2000)

10. Y. Kooy-Winkelaar, M. van Lummel, A. K. Moustakas, J. Schweizer, M. L. Mearin, C. J. Mulder, B. O. Roep, J. W. Drijfhout, G. K. Papadopoulos and J. van Bergen: Gluten-specific T cells cross-react between HLA-DQ8 and the HLA-DQ2α/DQ8β transdimer. *The Journal of Immunology*, 187(10), 5123-5129 (2011)

11. Y. van de Wal, Y. M. Kooy, P. van Veelen, W. Vader, S. A. August, J. W. Drijfhout, S. A. Peña and F. Koning: Glutenin is involved in the gluten‐driven mucosal T cell response. *European Journal of Immunology*, 29(10), 3133-3139 (1999)

12. C.-Y. Kim, H. Quarsten, E. Bergseng, C. Khosla and L. M. Sollid: Structural basis for HLA-DQ2-mediated presentation of gluten epitopes in celiac disease. *Proceedings of the National Academy of Sciences*, 101(12), 4175-4179 (2004)

13. M. Bodd, C. Y. Kim, K. E. Lundin and L. M. Sollid: T-cell response to gluten in patients with HLA-DQ2. 2 reveals requirement of peptide-MHC stability in celiac disease. *Gastroenterology*, 142(3), 552-561 (2012)

14. H. Arentz-Hansen, B. Fleckenstein, Ø. Molberg, H. Scott, F. Koning, G. Jung, P. Roepstorff, K. E. Lundin and L. M. Sollid: The molecular basis for oat intolerance in patients with celiac disease. *J PLoS Med*, 1(1), e1 (2004)
